# New Insights into Hematopoietic Stem Cell Expansion to Stimulate Repopulation of the Adult Blood System for Transplantation

**DOI:** 10.3390/life12050716

**Published:** 2022-05-11

**Authors:** Jiangying Xuan, Yingxia Liu, Jinhui Liu, Xiaoping Zeng, Hongmei Wang

**Affiliations:** 1 School of Basic Medical Sciences, Nanchang University, Nanchang 330006, China; xjy463399109@163.com (J.X.); welkin0120@foxmail.com (Y.L.); jinhuil2006@163.com (J.L.); zeng-xp@163.com (X.Z.); 2 Queen Mary School, Nanchang University, Nanchang 330006, China

**Keywords:** hematopoietic stem cells, in vivo expansion, ex vivo expansion, signaling pathway, microenvironment regulation, cultures

## Abstract

Successful engraftment of hematopoietic stem cells (HSCs) and progenitor cells (HSPCs) may be considered as a basis for the repopulation of the blood cells after transplantation in adults. Therefore, in vivo and ex vivo expansion of HSCs holds great promise for clinical applications. In this review, the mechanisms of HSC expansion will be discussed, considering the previous studies and works of literature. This is aimed to identify the signaling pathways that regulate HSC expansion and improve the application of engraftment in disease management. The following aspects will be included: (i) Stimulation of HSCs growth in vivo through gene regulation and cytokines activation; (ii) direct or indirect induction of HSC expansion by regulating signaling pathways; (iii) addition to assisting cells to help in the proliferation of HSCs; (iv) changing of living environment in the HSCs cultures via adjusting components and forms of cultures; (v) enhancement of HSC expansion by incorporating substances, such as extracellular vesicles (EVs), UM171, among others. In this review, recent new findings that provide us with new insights into HSC expansion methods have been summarized. Furthermore, these findings will also provide more possibilities for the development of some novel strategies for expanding and engrafting HSCs applied for treatments of some hematopoietic disorders.

## 1. Introduction

HSCs are multipotent progenitor cells that can differentiate into all types of blood cells, which play an important role in the blood system. HSCs are kept dormant in the bone marrow (BM) under normal conditions to preserve their long-term self-renewal potential and prevent stem cell exhaustion. A strictly regulated local niche exists in BM, which is composed of different cells, cytokines and extracellular matrix, and regulates the self-renewal, resting state, proliferation and differentiation of HSCs [1]. The niche is perivascular, created partly by mesenchymal stromal cells and endothelial cells, and often, but not always, located near trabecular bone [2]. Regulatory signals of niches are derived from surrounding cells or their cytokines, and physical factors, such as oxygen shear stress, contractile forces and temperature. In homeostasis, most HSCs are in a resting state. When exposed to chemotherapy injury, infection and other stimuli, HSCs are activated and begin to proliferate and differentiate in large quantities in response to the stimulation of some infectious stress, such as interferon-mediated signaling [3]. In the process of differentiation, HSCs first lose their self-renewal ability, followed by a gradual loss of pluripotency, and finally, they become specific functional mature cell types, such as erythrocytes, platelets, granulocytes, dendritic cells (DCs), B cells, T cells, natural killer (NK) cells. After BM transplantation, only the successful homing of HSCs can ensure that the long-term repopulation of the blood system is initiated. Thus, improving low efficacy in the engraftment of clinical hematopoietic transplantation has become an important challenge in the treatment of previously incurable hematopoietic diseases over the past few decades [4]. For years, available sources of HSCs are the BM, circulating peripheral blood and umbilical cord blood (UCB). UCB is considered to be an attractive source of HSCs for patients without HSCs phenotypically matched or unrelated donors [5]. Phenotypically, these cells show a CD34+, CD38−, CD90+, Lin−, and CD45RA− pattern as surface markers. Recent studies have shown that genetically modifying the surface marker expression profile of HSCs can regulate HSC expansion either positively or negatively [6]. The hematopoietic transplantation outcomes also largely depend on the number of cells infused. The way to increase the number is through efficient ex vivo expansion and the removal of the inhibition environment to expand HSCs in vivo [7]. Thus, successful in vivo expansion of HSCs would significantly benefit the therapies of some hematological diseases, such as aplastic anemia, cancers—particularly leukemia—and organ transplantation, and provide a better understanding of molecular mechanisms associated with differentiation and the growth of HSCs. HSC maintenance factors and the characterization of the BM niche in vivo have been recognized gradually by substantial efforts in clinical practice and animal experiments. However, the inability to efficiently expand HSCs ex vivo and the poor stability of expanded HSCs ex vivo greatly limit the application of HSCs transplantation in clinical practice [8,9]. Uncovering the cellular and molecular mechanisms underlying the regulation of HSCs expansion that could be regulated, therefore, has facilitated the development of new strategies for ex vivo HSCs self-renewal [10], and significant advances have been made to expand HSCs. In this review, we summarize the recent findings that elucidate the basic mechanisms of induced factors and specific HSC expansion techniques to improve HSCs engraftment and reduce some economic costs.

## 2. In Vivo Expansion of HSCs: The Disorders in the Hematopoietic System with Its Relevant Regulation and Potential Mechanisms

The in vivo expansion of HSCs and HSPCs is crucial to the treatment of several diseases. Although in vivo expansion of HSCs has been considered to be a unique strategy for treating diseases for years, the lack of expanding rate and survival rate in HSCs limits its application. Some diseases contribute to the failure of BM and impair the expansion of HSCs. Therefore, understanding the mechanisms of the diseases may promote the reestablishment of HSC expansion in vivo. Further, in vivo HSC expansion could be a future direction for research, owing to its convenience in the hematopoietic field. Here, we focus on the useful methods of expanding HSCs and HSPCs in vivo as the potential treatment of some hematopoietic diseases.

Anemia is a disease caused by the lack of erythrocytes. It can be categorized into various types, including aplastic anemia and Fanconi anemia (FA). The standard method for treating a hematological genetic disorder involves repairing the defective gene, possibly through gene therapy (GT), or by replacing the patient’s hematopoietic stem cells (HSCs) with normal HSCs from a healthy matching donor or with the patient’s genetically engineered HSCs that have been genetically engineered. Thrombopoietin (THPO) activates signaling by the myeloproliferative leukemia protein (MPL) on HSCs. Both thrombopoietin and MPL are required for HSC maintenance, as well as for megakaryocyte and platelet production [11]. The THPO receptor agonist, eltrombopag, has been considered as one of the treatments for aplastic anemia. While THPO directly stimulates the expansion of HSPCs, it also keeps HSCs in a quiescence state to build up a reserve pool of HSCs in the BM [12]. Moreover, THPO activates mitochondria and induces HSCs differentiation to megakaryocyte/platelet lineage cells. FA is a genetic disease characterized by BM failure and is more likely to progress into leukemia. It is demonstrated that the blockage of the non-homologous end-joining activity of DNA-PKcs prevented DNA damage-induced expansion of FA pre-leukemic HSCs [13].

**Figure 1:**
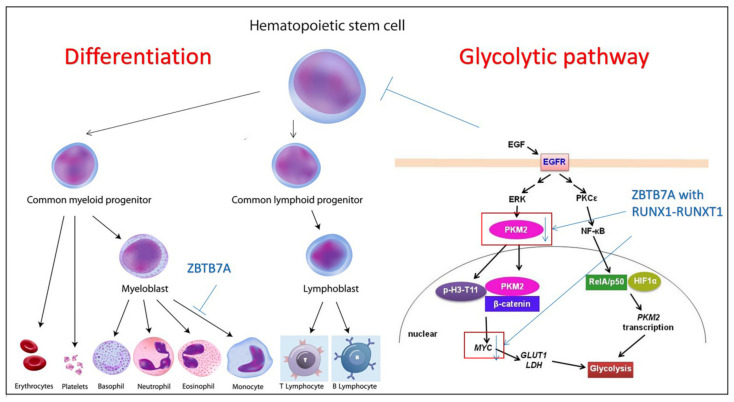
ZBTB7A acts on the process with HSCs’ differentiation and glycolytic pathways to regulate HSC expansion. In HSC differentiation, ZBTB7A mutations disturb myeloid HSCs and HSPC differentiation. In the glycolytic pathway, ZBTB7A with RUNX1-RUNX1T1 fusion gene can downregulate MYC and PKM2 to promote glycolysis and sensitize leukemic blasts.

Expansion of HSCs in vivo, directly or indirectly, is one of the most common ways for potential molecules to treat diseases. For example, high-throughput screening revealed that butyzamide, an eltrombopag prototype, can not only support an increase in the number of platelets but also trigger the expansion of HSPCs. Butyzamide can induce the multiplication of both megakaryocytes and HSCs in vivo by increasing the proportion of both human CD45+ cells and human CD34+CD38-cells, suggesting that butyzamide and other molecules with similar bioactivities could be developed as a potential treatment for aplastic anemia [14]. Another pathway is the ZBTB7A effects on the expansion of HSCs. The ZBTB7A mutation may collaborate with the RUNX1-RUNX1T1 fusion gene in two ways and could be a new target to treat acute myeloid leukemia (AML) (Figure 1). One way is that relevant ZBTB7A mutations in t (8;21) AML can contribute to perturbed myeloid differentiation with blockage of the monocytic differentiation to promote granulopoiesis; they can also disturb the differentiation of HSCs and HSPCs. Another way is that these mutations influence the growth regulation and metabolism of the ZBTB7A and RUNX1-RUNX1T1 fusion genes. ZBTB7A mutations are associated with the downregulation of MYC and PKM2, a key regulator and a key enzyme of the glycolytic pathway, respectively. Therefore, loss of ZBTB7A can enhance glycolysis and sensitize leukemic blasts to metabolic inhibition with 2-deoxy- D-glucose. Moreover, ZBTB7A expression in t (8;21) cells can result in a cell-cycle arrest, which mainly acts on G0/G1 arrest of the S phase, which can be imitated by blockage of glycolysis. Therefore, loss of ZBTB7A may facilitate the onset of t (8;21) AML, and that RUNX1-RUNX1T1-rearranged leukemia might be mimicked by glycolytic inhibitors because lack of ZBTB7A can enhance glycolysis [15].

Further, the relationship between CD166+ lineage- Sca-1+ c-Kit+ (LSK) cells and CD166+ osteoblasts (OBs) can expand colony-forming units by means via CD166–CD166 interaction [16]. Previous studies showed that mitochondria dysfunction-mediated signaling pathways were downregulated while metabolism and unfolded protein responses were upregulated in CD166- LSK cells [17]. Moreover, the CD166 knockout mouse changed the RNA profile of cultured cells, such as HSCs, compared to the wild-type mouse. For example, the RNA-seq showed that the upregulated genes in CD166- HSCs were general myeloid progenitors (GMP)-related genes and all GMP-specific genes were not downregulated. Meanwhile, the downregulated genes were mostly HSC-specific and none of the HSC-related genes were upregulated. Thus, CD166+ cells could be a crucial factor in maintaining stem/progenitor cell function [18].

Numerous studies have explored HSC expansion in vivo. A study evaluating the relationship between THPO and the cytokine signaling inhibitor lymphocyte adaptor protein (LNK), indicated that THPO and LNK act as opposing regulators of HSC expansion [19]. LNK decreases THPO-mediated HSC expansion. On the other hand, THPO is considered to be a positive regulator, stimulating HSC expansion and limiting the level of stem cell factors (SCFs). LNK cells show increased sensitivity to cytokines and altered activation of the Ras/MAPK pathway in response to some cytokines. The expression of a mutant LNK abolishes the binding capacity of the protein, increasing the number of CD451 cells [20]. However, the activity of THPO signaling is strictly regulated by LNK [21]. The chemical components of the cocktail can also play an important biological role in the biological field by overexpressing transcription factors. Mouse fibroblasts can be reprogrammed into hemogenic cells by lineage tracing analysis without inducing exogenous genes. Progressively, they can develop into HSCs by adding the chemical cocktail. The role of the chemical cocktail is to activate key transcription factors, thus reprogramming the differentiated hematopoietic cells back into HSPC-like cells. Chemical cocktails activating Sox2 might enhance fibroblasts with hematopoietic potential. Moreover, some chemical cocktail compounds can promote HSPC expansion. For example, valproic acid (VPA), a histone deacetylases inhibitor, has been reported to promote HSPC proliferation, and this effect is further enhanced by the addition of the GSK3β inhibitor [22]. HSCs are crucial in maintaining the repopulating potential in the fetal liver. The liver has multiple functions, including detoxification, secretion of digestive hormones, and regulation of electrolyte balance. It is proved that the fetal liver repopulating unit contributes to the maintenance of the competitive repopulating unit and stem cells [23].

It was found that the HSCs with over-expression of *HoxB4* had a greater ability to expand than those that were not transduced with the *HoxB4* gene. However, the mechanism of how *HoxB4* expands HSCs in vivo is not known and the number of expansions is also limiting. In vitro expansion by *HoxB4* can offset these disadvantages by adding parameters to control the microenvironment, which is discussed in a later part. Hox gene can play a role not only in the in vivo expansion but also in the in vitro expansion of HSCs [24].

Moreover, myeloid expansion can be enhanced by regulating the microenvironment, which is composed of HSCs and non-hematopoietic cells [25]. Remodeling of BM HSC niches can affect HSC expansion and premature aging, based on BM growth, which may be associated with the production of inflammatory cytokines [26]. This can be achieved by increasing BM noradrenergic innervation to promote b2-adrenergic-receptor (AR)-interleukin-6-dependent megakaryopoiesis, reducing b3-AR-Nos1 activity. Increased b2-AR activity enhances IL-6-dependent myeloid differentiation [27]. Meanwhile, decreased b3-AR-Nos1-NO can reduce endosteal niches and increase central niches. Thus, the regulation of the microenvironment can change the survival condition of HSCs, and the regulation goal is to expand HSCs [28].

## 3. Ex Vivo Expansion of HSCs

Although significant efforts have been made to characterize HSC maintenance factors through the characterization of the in vivo BM HSC microenvironment or niche, stable in vivo HSC expansion has previously been unattainable [8]. This is because the conditions that maintain primitive human repopulating cells and support their characteristics are poorly understood. In addition, in vivo HSC expansion may not affect treating diseases without identifying the exact targets in specific diseases [29]. Thus, in vitro culture of HSCs is considered as an important pathway applied in clinical diseases, such as gene therapy, tumor cell clearing, and stem/progenitor cell expansion [30]. The signaling pathways and mechanisms in HSC growth are explained, then, different aspects, such as genes, cell cultures and small molecules used in HSC expansion are also introduced.

### 3.1. Signaling Pathways Regulating HSC Expansion

**Figure 2:**
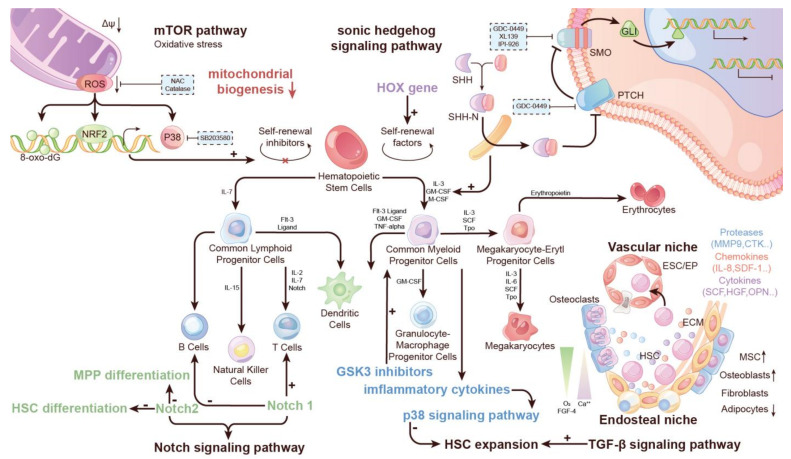
Overview of signaling pathways involved in HSC expansion. Shh, GSK3, TGF-b, Notch and Hox signaling pathways are the basic pathways that regulate HSC expansion via affecting cell differentiation and circumstances. Signaling pathways have both positive and negative effects on HSC amplification.

Previous studies have demonstrated that activating or inhibiting signaling pathways can regulate HSC growth, and thus control HSC engraftment and in vitro HSC expansion. These findings have inspired many subsequent studies on the identification, purification and characterization of signaling pathways in HSC expansion. Many signals have been demonstrated to have effects on the cell self-renewal, development and cell cycle (Figure 2).

For instance, Sonic Hedgehog (Shh) and bone morphogenetic protein-4 (BMP-4) could be effective in expanding HSCs and HSPCs [25]. Adding the Shh factor and some growth factors in the culture (FL, SF, G-CSF, IL-3 and IL-6) can enhance the recovery of repopulating cells of human cord blood CD34+ CD38-Lin-cells [24]. Nociceptor neurons drive G-CSF-induced HSC mobilization via the secretion of calcitonin gene-related peptides [31]. It has been demonstrated that over-activation of mTOR impairs HSC function and causes HSC depletion in vivo under various pathological conditions and during aging by repressing mitochondrial biogenesis and reactive oxygen species (ROS) [14]. Through decades of research, HSC expansion is influenced by both GSK3 and p38 signaling pathways. It has been discovered that a proper concentration of the GSK3 inhibitor and Wnt signaling enhance HSC expansion because of moderate levels of β-catenin activity in HSCs [32]. Such GSK3 inhibitor concentration can also activate myeloid cells to produce inflammatory cytokines, reducing HSC expansion, thereby stimulating the p38 signaling pathway. Therefore, blockage of p38-induced inflammatory cytokine signaling was required to guarantee HSC expansion, induced by a low concentration of the GSK3 inhibitor. A previous study demonstrated that both a moderate concentration of p38 inhibitor and GSK3 inhibitors synergistically increase the expansion of both murine BM HSCs and human UCB HSCs [33].

The transforming growth factor-β (TGF-β) signaling pathway is a crucial factor for the establishment of normal hematopoietic niches in BM. It plays a vital role in the lineage-specific expansion of mesenchymal stromal cells (MSCs), which are essential components of hematopoietic niches in the BM. MSCs can differentiate into OBs, adipocytes and chondrocytes in vitro under particular conditions, and TGF-β is a key cytokine to inhibit the differentiation of MSCs into OBs [34]. Thus, MSCs can determine HSC properties by interacting with cytokines, promoting the self-renewal and expansion of HSCs. For example, trombospondin-1 (TSP-1) can positively activate TGF-β signaling. TSP-1 also promotes MSCs proliferation and migration by platelet-derived growth factor (PDGF) protection against MSCs-derived protease. Trombospondin-2 (TSP-2) leads to the differentiation of MSC into chondrocytes through P38MAPK, PKC, ERK and Notch signaling pathways [3,35]. The importance of the TGF-β signaling pathway has been confirmed by knocking out Tgfbr2 in mesenchymal cells using a doxycycline- repressible Sp7 (osterix)-Cre trans-gene. Firstly, loss of TGF-β causes a decrease in OBs but an increase in CXCL12-abundant reticular (CAR) cells and adipocytes in the BM. CXCL12 is a chemokine that is required for HSC maintenance and HSC retention in the bone marrow HSCs commonly localize adjacent to Nes-GFP+ cells, and these cells express high levels of Scf and CXCL12 [2]. Secondly, TGF-β signaling is necessary for maintaining the characteristics of mesenchymal cells in the BM after birth under a steady-state microenvironment. Overall, TGF-β but not definitive mesenchymal stem/progenitor cells (MSPCs) play a vital role in the lineage specification of the fetal BM and are required for the establishment of normal hematopoietic niches in fetal and perinatal BM [36].

The Notch signaling pathway can be proved to mediate the differentiation of HSCs. It has been found that Notch1 can facilitate T-cell differentiation and inhibit B-cell differentiation [37]. Notch2 signaling affects HSC self-renewal by inhibiting the differentiation of HSCs to multipotent progenitors (MPP) and MPP differentiation to myeloid/monocytic (M) cell lineage [16].

More recent studies suggested that the activation of the Notch signaling pathway in HSPC self-renewal, by cytokine stimulation, can increase the expansion of HSCs, thus attracting considerable interest [25]. However, a fundamental disadvantage of all HSC-driven culture systems is the rapid generation of differentiating cells and their secreted inhibitory feedback signals; therefore, automated control of inhibitory feedback signaling can be very important in promoting the expansion of HSCs [38]. To establish a specific HSC-driven culture system, Elizabeth Csaszar et al. have employed a controlled fed-batch media dilution approach called the fed-batch strategy. The fed-batch strategy is important in determining the mode of action of the aryl hydrocarbon receptor antagonist SR1 and the TAT-HOXB4 protein to efficiently expand HSCs. The result has indicated that application of this system to human cord blood cells presented a rapid (12-day) 11-fold increase in HSCs with self-renewing and multi-lineage repopulating ability, which underscores the marked improvements that the control of feedback signaling can offer a primary stem cell culture and demonstrates a clinically relevant rapid and relatively low culture volume strategy for ex vivo HSC expansion [7].

### 3.2. Related Genes in Regulation of HSC Expansion

Hox genes encode a large family of transcription factors which are considered the homeodomain [5]. Hox transcription factors are essential regulators of primitive hematopoietic cell proliferation and differentiation, especially *HOXB4*, which is particularly characterized as a positive regulator of HSC self-renewal but does not affect HSC differentiation and cell transformation. Cultures of non-transduced and *GFP*-*HoxB4*-transduced murine BM cells both showed a large HSCs loss over 10–14 days. The results demonstrated that cultures of *HOXB4*-transduced cells might rapidly achieve highly polyclonal HSC expansions. In contrast, controls achieved a less fold net HSC increase. Mechanistically, *HoxB4* fusion protein is taken up by the CD34 cells to increase the long-term repopulating cells [25]. Consequently, these HSCs retained full lympho-myeloid repopulating potential and increased the ability of HSC in vivo regenerative potential, suggesting the potential of available ex vivo expansion of HSCs without functional impairment [39].

Another Hox-related gene that can be fused with HSCs to increase expansion is Nucleoporin98-Homeobox (NUP98-Hox) fusions. A previous study explored whether *HoxB4* could induce a remarkable expansion of HSCs in culture and extend this to other Hox genes and, thus, further analyzed the Hox sequence requirements to achieve this effect. The fusions consist of the N-terminus of NUP98, which summarizes a region of multiple phenylalanine-glycine repeats that may be considered as a transcriptional co-activator through binding to CBP/p300 and the C-terminus of the Hox gene product, including the intact homeodomain and a variable portion of the flanking amino acids. For example, the main type of the gene fusion, the NUP98-*HoxA10* fusion gene, could block terminal differentiation, contributing to the sustained output of cells with a ‘primitive’ phenotype, which showed more potency in a short-term stimulation of HSC expansion [40].

Forkhead box (Fox) genes can encode many transcription factors. It has been demonstrated that FoxM1 is essential for quiescence maintenance in HSCs by inducing the expression of Nurr1, which preserves quiescence of HSCs via inducing the expression of p21 and p27. In addition, deletion of FoxO1, FoxO3, and FoxO4 increases cell cycling and apoptosis in quiescent HSCs via alteration of the expression of their target genes, including cyclin G2, cyclin D, p21, and p27 [41,42].

Several clinical applications have shown that the surface cell markers of ex vivo-expanded HSCs are essential to their purification and analysis. Different stem cell markers exist in different amounts and in different stages in cultured HSCs, which show different surface phenotypes in HSC cultures. For instance, freshly isolated HSCs in the defined cultures were positive for the stem cell markers Sca-1, Kit and CD31, and receptors for an insulin-like growth factor (IGF-2). Interestingly, prion protein and Tie-2 were expressed on freshly isolated HSCs but not on cultured HSCs. The other two HSC markers, Endoglin and Mpl, were expressed only on a portion of cultured HSCs. The result indicated that the surface phenotype of ex vivo-expanded HSCs can be distinguished from that of freshly isolated HSCs. One study has identified that Integrin-a3 (ITGA3) as a marker of cultured human HSCs, and the pyrimidoindole derivative UM171 can effectively expand cord blood cells. ITGA3 is a reliable marker of cultured human long-term hematopoietic stem cells (LT-HSCs) [20]. Of note, the plasticity of surface cell markers may not be associated with repopulation capability.

### 3.3. Assisting Cells Affecting HSC Expansion

BM HSCs and HSPCs can age prematurely after transplantation, which may impair function or lead to functional loss. Therefore, it is necessary to expand sufficient HSCs and prevent early senescence before engraftment. Some cells can promote the expansion of HSCs or play critical roles in regulating HSCs while they are co-cultured with HSCs ex vivo. Thus, assisting cells are an important component for sustaining the culture state to create a microenvironment for the expansion of HSCs.

The fetal liver can play an important role in tissue engineering, especially in liver regeneration. This can be also crucial to hematopoiesis [43]. The liver is the main site of hematopoiesis in the human embryo. In addition, numerous studies showed that fetal liver cultures provided reciprocal support for cells’ development when HSCs and other cells are co-cultured. Human fetal liver sinusoidal endothelial cells (hFLSECs) may play a vital role in HSC expansion. A study has demonstrated that human fetal liver sinusoidal endothelial cells which expressed the E4orf1 gene (hFLSECs-E4orf1) can efficiently amplify HSPCs and HSCs ex vivo, and the amplified cells can be transplanted in vivo. E4orf1 gene has a long-live phenotype and provides “anti-apoptotic” signals in HSCs [44]. Moreover, hFLSECs can highly express hematopoiesis-related growth factors and Notch receptors, indicating Notch signaling pathway activation. Consequently, hFLSECs may provide a microenvironment to support HSC expansion and growth factor activation [45]. Another cell that can effectively expand HSCs is the fetal liver stromal cell (StroCs). A previous study has demonstrated that the high expression of Shh and Wnt in StroCs can enhance HSPC amplification, and the effect can be inhibited by using the Shh inhibitor cyclopamine to suppress Shh signaling. However, adding soluble Shh-N can promote HSC expansion by activating Wnt signaling [46].

Here, we discuss the positive effect of alternative (M2)-polarized macrophages (M2- MFs) and the negative effect of classical (M1)-polarized macrophages (M1-MFs) in the self-renewal and proliferation of HSCs from mouse BM in vitro. The opposite effect of macrophages may be induced by the differential expression of nitric oxide synthase 2 (NOS2) and arginase 1 (Arg1). M1-MFs are prone to induce inflammatory factors, ROS and nitric oxide, which are associated with the role of MFs in fighting against microbial infection and cancer. M2-MFs have an anti-inflammatory function. Because M2-MFs can promote the expansion of HSCs, when co-cultured with hUCB CD34+ cells with M2-MFs, an increase in CD34+ cells and severe combined immunodeficiency (SCID) mouse-repopulating cells can be observed [10].

Human brain endothelial cells (HUBECs) can affect the phenotype of human HSCs in ex vivo culture to support the expansion of cells which can repopulate non-obese diabetic/SCID mouse-repopulating cells (SRCs). It was found that HUBECs can support the expansion of CD34+CD38-cells because the downregulated CD38 surface expression makes it difficult to measure HSCs content in ex vivo culture conditions [47]. Therefore, recently developed SRCs can provide a way to characterize human repopulating cells and factors that regulate the environment [30]. Meanwhile, non-contact HUBECs cultures and THPO, SCFs and macrophage colony-stimulating factor I receptor (Fms), such as tyrosine kinase (Flt-3) ligand can play additional roles in CD34+CD38-cells, with increased activity of SRCs [48]. Moreover, compared with the CD34-CD38- population after culture, CD34+CD38- subtype has more SRCs, which shows that HSCs can be identified by phenotype after culture expansion, and HUBECs-related soluble factors mediate an effective expansion of HSCs [49,50]. Endothelial cells are also related to the perivascular niche. A study found that the conditional deletion of the gene that encodes the gp130 cytokine receptor in endothelial cells can contribute to bone marrow hypocellularity and a reduction in HSC numbers. It has been suggested that E-selectin is exclusively expressed by endothelial cells in the bone marrow, and deficiency of the gene that encodes it renders HSCs more quiescent and resistant to irradiation [51].

Nestin expression MSCs make up an essential HSC component. Nestin+ MSCs contain all the bone-marrow colony-forming-unit fibroblastic activity and can be expanded as non-adherent ‘mesenspheres’. The ‘mesenspheres’ can self-renew and expand in serial transplantations. Moreover, nestin+ MSCs are spatially associated with HSCs and adrenergic nerve fibers, and highly express HSC maintenance genes, which can trigger osteoblastic differentiation [52].

### 3.4. Components and Forms of Cultures Affect HSC Expansion

Mounting evidence suggests that the condition of a defined culture can significantly enhance HSC expansion capability. Surprisingly, compared with HSCs without altered culture, HSCs whose methods and the components of the culture are completely changed can be expanded rapidly [20].

A recent study discovered that a 3D culture in a zwitterionic hydrogel can expand phenotypically primitive CD34+ cord blood and BM-derived HSPCs, which achieved substantial expansion of LT-HSCs frequency. The expansion of HSCs was reached via the inhibition of HSPC differentiation and increased self-renewal caused by the 3D zwitterionic hydrogel culture because of an inhibition of excessive ROS production by suppressing O-related metabolism [53]. However, the culture condition, the zwitterionic characteristics of the hydrogel and the 3D format are both vital for the expansion [54].

Interestingly, the properties of growth factors may positively change the self-renewal and differentiation of stem cells [24]. Cronkite discovered that adult mouse marrow cells in culture could significantly expand with the stimulation of IL-11, FL, and SF, which can lead to the restoration of the ability of their lympho-myeloid cells. Moreover, the integrity of cellular properties can be affected by the types of receptors activated and the intensity and/or duration of the activation [19]. Miller and Eaves also proved the ability of IL-11, FL and SF to expand HSCs ex vivo, with a significant expansion of culture-initiating cells and myeloid colony-forming cells. Reconstituted stem cells from the BM of normal adults may not have the ability to proliferate in a semisolid medium [55]. Therefore, FL, SF and IL-11 are added to primitive fetal liver cells to synergize mitogenic activity and enhance the potency of stem cells. However, exposure of fetal liver cells to a combination of IL-11, FL and SF can lead to the loss of long-term repopulating ability, although it can stimulate HSC self-renewal divisions in vitro [1].

Moreover, the increase in the number of long-term cultures initiating cells and competitive repopulating units is very similar in several recent studies; however, the increase in colony-forming cells and total cell numbers can be far more rapid [56]. A simple serum-free culture system containing appropriate levels of SCFs, THPO, insulin-like growth factor 2 (IGF-2), and fibroblast growth factor-1 (FGF-1) was developed for BM HSCs. The result showed a significant expansion in numbers of long-term HSCs compared to a short-term culture of total BM cells [3].

An albumin-free system can also support the expansion of HSCs. Because a high level of THPO with a low level of stem-cell factor and fibronectin can lead to an HSC self-renewal albumin-free system, the serum albumin can be considered as the contaminants in HSC cultures [57]. A culture with polyvinyl alcohol (PVA), which is used to replace serum albumin, could induce the secretion of the pro-inflammatory cytokines. However, the culture may lead to considerable heterogeneity in the self-renewal capacity of HSCs ex vivo [8]. Because of the different relative or absolute cytokine concentrations, the potency of cellular properties can be affected, which warrants further investigation in the future [5].

For years, hUCB has been clinically recognized as an available source of HSCs for efficient hematopoietic engraftment [6]. Compared with stem cells, the main advantages of hUCB are easier acquisition, lower risk from the donor, dramatically reduced risk of infection, and the more rapid availability of samples. [58]. However, UCB transplantation therapy is limited by low numbers of HSCs per unit of UCB [33], and there is a delay in uHSCs’ attachment and homing into the natural niche owing to the incomplete carbohydrate chain [59].

A previous study explored approaches to expand fucosylated UCB HSCs. The results showed that a selectin-coated scaffold could be a supportive structure when using nano-topography. The selectin-coated scaffold can act as a basic surface in cell–cell interaction to stimulate more uHSC potential. P-selectin, which is a transmembrane glycoprotein, is expressed by endothelial cells and platelets. Moreover, due to the difficulty of homing to the natural niche of uHSCs, a nano scaffold was used to mimic the natural niche of uHSCs. By coating the scaffolds with selectin, receptors for the fucosylated cell glycan ligand adhesion and survival of HSCs can be created. Based on these results, selectin-coated poly l-lactic acid scaffolds could be applied to hematological tissue engineering and HSCs transplantation using human-fucosylated cells. Thus, this strategy may play an important role in hematological disorders in the future [59].

### 3.5. Other Factors in HSC Expansion

**Figure 3:**
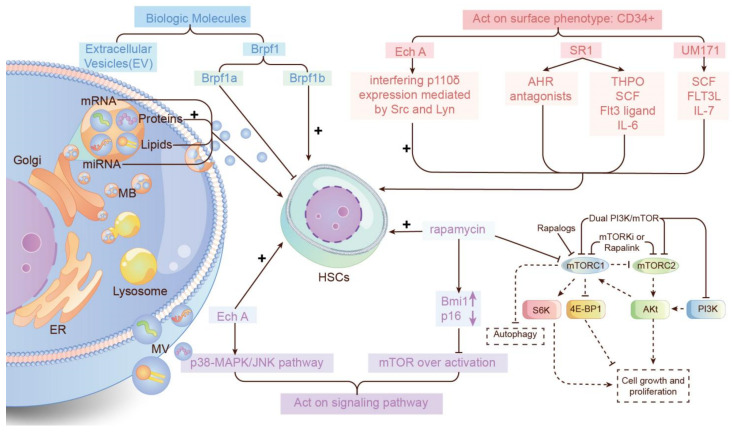
Overview of substances that expand HSCs in vitro. Substances can be classified into 3 categories based on their action on HSC expansion: act by biologic molecules, act on surface phenotype (CD34+), and act on signaling pathway. Biologic molecules act by producing bioactivators to create necessary substances to expand HSCs. CD34+ is the typical phenotype that stimulates HSC expansion. The signaling pathway has been previously discussed.

Besides the above-mentioned factors, other factors can also affect HSC expansion by influencing the microenvironment of HSCs, such as proteins, cytokines or regulation signaling pathways (Figure 3).

It was shown that BM-derived MSCs can improve the expansion of HSCs through paracrine activity, and some can be mediated by EVs. The EVs can transport some proteins, lipids and nucleic acids to the HSCs. Therefore, they can be considered “cell-free biologics” to expand HSCs in vitro [58].

UM171, an agonist pyrimidoindole derivative, is one of the most effective small molecules that can significantly expand hPSCs. UM171-expanded cord blood cell is feasible and safe and allows for the use of small single cords without affecting engraftment. Further, UM171 can act as a new substance to expand HSCs without the risk of chronic graft-versus-host disease (GVHD) and relapse [32,33]. Moreover, UM171 plays an important role in treating lymphoma [60,61]. A study explored the culture of hPSC-HPs in HSC expansion conditions. In the presence of THPO, SCF, FLT3L, IL3 and IL-6, the UM171-expanded is a specialized phenotype CD34+CD41aloCD45+ and was enriched in granulocytic progenitors (G-CFCs) [62]. However, in the cultures with SCF, FLT3L and IL7, UM171 selectively amplified the CD34+CD45+CD7+ phenotype with NK cells [60]. It is suggested that UM171 can act on one specific phenotype with different expansion conditions, although the mechanisms remain elusive. One theory demonstrated that UM171 can upregulate HSCs-specific, mast cell-specific and non-canonical Wnt signaling-related genes. Meanwhile, it inhibits the expression of erythroid, megakaryocytic genes and inflammatory mediated chemokine, thus inhibiting erythroid and megakaryocytic differentiation [60]. Another theory described the critical balance of UM171, whereby UM171 expands HSCs by building up a balance between pro- and anti-inflammatory mediators of self-renewal, which is based on NFKB activation and protein C receptor-dependent ROS detoxification, respectively [63]. In this balance, endothelial protein C receptor (EPCR) is a crucial component that inhibits ROS accumulation and prevents function loss of the stem cell [64].

Echinochrome A (ech A) is a substance that can significantly expand the CD34+ cells to induce HSPC expansion by interfering with the expression of p110δ mediated by Src and Lyn. Therefore, the production of ROS is inhibited and p38-MAPK/JNK pathway is activated. The CD34+ can be expanded [65]. It is known that one of the most important signaling molecules required for HSC homing is the C-X-C chemokine receptor 4 (CXCR4). Niche, containing high levels of calcium, suggests that HSCs are sensitive to Ca^2+^. Studies have proved that mouse HSCs have very few free cytosolic Ca^2+^ [66]. The stimulation with stromal cell-derived factor 1 (SDF-1) activates CXCR4, inducing an inositol trisphosphate (Ip3) mediated release of Ca^2+^ from the endoplasmic reticulum via Ip3 receptors (Ip3R) with transient elevation of Ca^2+^ concentration [67,68]. Therefore, some researchers use PGE2 inhibitors or CXCR4 expression enhancers to improve the engraftment and increase the Ca^2+^ concentration by enhancing the ability of the lower numbers of stem cells available in a CB unit to home to the stem cell niche [7]. Copper chelator—TEPA and PGE2 can also promote HSC expansion, however, their mechanisms of action are still not clear. An unbiased screening has demonstrated that StemRegenin1 (SR1) can enhance the ex vivo expansion of CD34 cells, leading to a 50-fold increase. SR1 is activated by aryl hydrocarbon receptor antagonists [9]. Mechanically, this involves the expression of HES-1, PU.1, and C/EBP-b catenin [25]. However, it becomes ineffective when SR1 adds to the culture alone. SR1 needs cytokines to play roles: THPO, SCF, Flt3 ligand and IL-6. Therefore, the discovery of SR1 may be a milestone in proving that small synthetic chemicals can be applied to recognizing the signaling pathways to expand HSCs in clinical practice [69].

The mTOR acts as a regulator of in vitro self-renewal of HSCs and rapamycin can provide a new way to promote the HSC expansion by inhibiting the activation of mTOR. However, the condition of HSCs may be related to the extent of mTOR activation, which can be regulated by rapamycin. A time-dependent activation can cause HSCs enrichment, but the over-activation of mTOR may lead to LSK senescence and not apoptosis [70]. Therefore, rapamycin can inhibit the over-activation of mTOR to inhibit the senescence of LSK cells, which can expand HSCs. Furthermore, the reason that the rapamycin can amplify HSCs may be attributed to the upregulation of Bmi1 and downregulation of p16 mediated by rapamycin [71]. One previous study demonstrated that somatic mutations of ASXL1 are frequently detected in age-related clonal hematopoiesis (CH). This kind of mutation can also interact with the Akt/mTOR pathway. The activation of Akt and mTOR signaling can be induced by phosphorylation of Akt and S6, respectively. This process may occur in young ASXL1 mutant knock-in HSPCs. However, the over-activation of Akt/mTOR signaling has been linked to impaired hematopoiesis in aged ASXL1 mutant knock-in mice [72].

Angiopoietin-like protein is a family that has been shown to expand HSCs ex vivo and, hence, might be a milestone in the field of stem cell biology. To measure the self-renewal ability of SRCs, the SCID-SRCs are transplanted into the non-obese diabetic SCID (NOD/SCID) mice. Results of the study showed that a serum-free culture including SCF, THPO and FGF-1 or Flt3-L cannot clearly amplify SRCs present in human cord blood CD133 cells [73]. However, when either angiopoietin-like protein 5 or IGF-binding protein 2 is included in the experiment, the expansion of HSCs becomes significant. Therefore, this can support about a 20-fold net expansion of repopulating human cord blood HSCs. It has been reported that Angptl5, Angptl 2 and Angptl3 can also stimulate HSCs expansion because of their coiled-coil domain [74]. A previous study has also reported the function of Angiopoietin-like 5 (Angptl5). It was shown that when using soluble growth factors of Angptl5, IGFBP2 and pleiotrophin, the number of HSPCs can be significantly enhanced ex vivo [25]. Moreover, it has been revealed that the interaction between tyrosine kinase Tie-2 and its ligand angiopoietin-1 can maintain the properties of immature progenitors [5,56].

The Brpf1 is another protein that can regulate the expansion of adult hematopoietic stem and HSPCs, hence the specific isoform can act differently in the expansion of HSCs. A previous study uses nine bromodomain inhibitors to investigate the relationship between Brpf1 and HSCs. It was found that the Brpf1 isoforms—Brpf1b—promoted the expansion of HSPCs. On the other hand, it has been reported that Brpf1a caused quiescence of HSPCs and led to the decreased expansion of HSPCs [75].

A chromatin protein, HMGB2, which binds to the Lxn promoter, has a significant influence on the transcriptional regulation of Lxn. Knockdown of HMGB2 increases Lxn expression, which suggests that Lxn is one of the downstream targets of HMGB2. Moreover, the depletion of HMGB2 decreases the number of functional HSCs by promoting apoptosis and reducing proliferation. Thus, the genetic and epigenetic regulation of Lxn transcription can be applied in HSC expansion [76].

Glutathione peroxidase 4 (GPX4) is a critical suppressor of lipid peroxidation and ferroptosis. A study found that HSCs and HSPCs lacking GPX4, accumulated lipid peroxidation and underwent ferroptosis in vitro. α-Tocopherol is the main component of vitamin E, which was proved to resist the GPX4-deficient HSPCs from ferroptosis in vitro. Furthermore, increased levels of lipid peroxidation and cell death indicated that HSPCs undergo ferroptosis. Collectively, GPX4 and vitamin E cooperatively maintain lipid redox balance and prevent ferroptosis in HSPCs [77].

Further, the β2 integrin family is critical for HSPC function under stress. Integrins containing α4, α6, and β7 subunits regulate HSPC homing. Integrin αvβ3 plays a role in maintaining the quiescence of HSPCs. CD11α has been shown to be important for lymphocyte progenitor development, but not for HSPCs. CD11c deficiency was associated with increased apoptosis and a significant loss of HSPCs in sepsis and bone marrow transplantation [78].

## 4. Reprogramming of HSCs

There are two ways to reprogram HSCs. One way is to expand the number of HSCs by reprogramming the senescent HSCs or another lineage of adult cells, changing them into the young HSC state. The other way is to reprogram HSCs into pluripotent stem cells so that they can be used to differentiate particular HSCs.

With more youthful bone marrow cellular composition and an improved regenerative capacity, HSCs repopulation can be easier and more applicable in a transplant setting. Moreover, aging is related to the functional decline of HSCs; reprogramming HSCs, including the redistribution of DNA methylation and reductions in H3K27ac, H3K4me1 and H3K4me3, can prevent the risks of age-related leukemia [79]. Nicotinamide riboside (NR), a form of vitamin B3, can restore youthful metabolic capacity by regulating mitochondrial function, such as reducing the expression of nuclear-encoded metabolic pathway genes, damping mitochondrial stress and decreasing mitochondrial mass and network size [80]. A series of transcription factors, Gata2, Gfi1b, cFos and Etv6, can efficiently activate the 34/H2BGFP reporter and induce endothelial-like precursor cells with the subsequent appearance of HSCs. Moreover, the precursor cells express a human CD34 reporter, Sca1 and Prominin1, which are the origin of HSCs [81]. A study has found that seven transcription factors (ERG, HOXA5, HOXA9, HOXA10, LCOR, RUNX1 and SPI1) were able to convert haemogenic endothelium into HSCs and HSPCs that engraft myeloid, B and T cells in primary and secondary mouse recipients [82]. Meanwhile, data analysis showed that using a placenta-derived cell medium can reprogram CD34+ hematopoietic stem cells to induce pluripotent stem cells [83].

Production of blood stem cells from reprogrammed adult cells is notoriously difficult. It emerges that a supportive microenvironment may be crucial for their efficient generation [84]. Sandler kept HSCs on a feeder-cell layer that mimics the HSC niche. The feeder cells, called E4ECs, were endothelial cells engineered to overexpress an adenoviral gene, E4ORF1, that promotes HSC survival [85]. Another study has reported the transformation of mature white blood cells from mice into engraftable HSCs that can form all blood-cell lineages. Reprogramming was accomplished with six transcription factors (Runx1t1, Hlf, Lmo2, Prdm5, Pbx1 and Zfp37), and the cells were matured in vivo to generate iHSCs [86].

## 5. Conclusions

In conclusion, hematopoietic transplantation is currently a major method for the treatment of several blood-related diseases, such as aplastic anemia, leukemia and organ transplantation. Therefore, the proliferation of HSCs can be important because it is related to the repopulation of blood cells after transplantation. In this review, we have concluded that HSCs can be expanded in many different ways, both in vivo and in vitro. Therefore, this can be a milestone for both clinical practice and academic research. The low success rate of cord blood engraftment is a problem in clinical fields. If the HSCs and HSPCs can be expanded effectively, then a dramatic increase in efficacy after transplantation can be realized. Therefore, the difficulties that there is an insufficient number of HSCs in cord blood engraftment and the inability to sustain the provision of HSCs may also be resolved. The problems are overcome by acting on the signaling pathways, changing gene surface markers, and discovering some substances or cells in the culture to support the condition or a microenvironment for HSCs expansion. However, there are also some limitations in the new materials, including that most of the factors cannot expand HSCs alone without the addition of other factors which may be harmful to the human body. Therefore, there is an urgent need for a new strategy to explore and resolve these problems and, thus, effectively expand the HSCs.

## Data Availability

All data are available in the manuscript.

## References

[1] Cha S.Y., Choi Y.H., Hwang S., Jeong J.Y., An H.J. (2017). Clinical impact of microRNAs associated with cancer stem cells as a prognostic factor in ovarian carcinoma. J. Cancer.

[2] Morrison S.J., Scadden D.T. (2014). The bone marrow niche for haematopoietic stem cells. Nature.

[3] Kaur S., Sehgal A., Wu A.C., Millard S.M., Batoon L., Sandrock C.J., Ferrari-Cestari M., Levesque J.P., Hume D.A., Raggatt L.J. (2021). Stable colony-stimulating factor 1 fusion protein treatment increases hematopoietic stem cell pool and enhances their mobilisation in mice. J. Hematol. Oncol..

[4] Girotra M., Trachsel V., Roch A., Lutolf M.P. (2020). In vivo pre-instructed hscs robustly execute asymmetric cell divisions in vitro. Int. J. Mol. Sci..

[5] Hofmeister C.C., Zhang J., Knight K.L., Le P., Stiff P.J. (2007). Ex vivo expansion of umbilical cord blood stem cells for transplantation: Growing knowledge from the hematopoietic niche. Bone Marrow Transplant..

[6] Derakhshani M., Abbaszadeh H., Movassaghpour A.A., Mehdizadeh A., Ebrahimi-Warkiani M., Yousefi M. (2019). Strategies for elevating hematopoietic stem cells expansion and engraftment capacity. Life Sci..

[7] Csaszar E., Kirouac D.C., Yu M., Wang W., Qiao W., Cooke M.P., Boitano A.E., Ito C., Zandstra P.W. (2012). Rapid expansion of human hematopoietic stem cells by automated control of inhibitory feedback signaling. Cell Stem Cell.

[8] Wilkinson A.C., Ishida R., Kikuchi M., Sudo K., Morita M., Crisostomo R.V., Yamamoto R., Loh K.M., Nakamura Y., Watanabe M. (2019). Long-term ex vivo haematopoietic-stem-cell expansion allows nonconditioned transplantation. Nature.

[9] Boitano A.E., Wang J., Romeo R., Bouchez L.C., Parker A.E., Sutton S.E., Walker J.R., Flaveny C.A., Perdew G.H., Denison M.S. (2010). Aryl hydrocarbon receptor antagonists promote the expansion of human hematopoietic stem cells. Science.

[10] Luo Y., Shao L., Chang J., Feng W., Liu Y.L., Cottler-Fox M.H., Emanuel P.D., Hauer-Jensen M., Bernstein I.D., Liu L. (2018). M1 and M2 macrophages differentially regulate hematopoietic stem cell self-renewal and ex vivo expansion. Blood Adv..

[11] Crane G.M., Jeffery E., Morrison S.J. (2017). Adult haematopoietic stem cell niches. Nat. Rev. Immunol..

[12] Guan Y., Hasipek M., Jiang D., Tiwari A.D., Grabowski D.R., Pagliuca S., Kongkiatkamon S., Patel B., Singh S., Parker Y. (2022). Eltrombopag inhibits TET dioxygenase to contribute to hematopoietic stem cell expansion in aplastic anemia. J. Clin. Investig..

[13] Chatla S., Wilson A.F., Pang Q. (2019). Inactivation of the NHEJ activity of DNA-PKcs prevents fanconi anemia pre-leukemic HSC expansion. Int. J. Stem Cells.

[14] Sakurai M., Takemoto H., Mori T., Okamoto S., Yamazaki S. (2020). In vivo expansion of functional human hematopoietic stem progenitor cells by butyzamide. Int. J. Hematol..

[15] Monte E.R., Wilding A., Leubolt G., Kerbs P., Bagnoli J.W., Hartmann L., Hiddemann W., Chen-Wichmann L., Krebs S., Blum H. (2020). ZBTB7A prevents RUNX1-RUNX1T1-dependent clonal expansion of human hematopoietic stem and progenitor cells. Oncogene.

[16] Capo V., Penna S., Merelli I., Barcella M., Scala S., Basso-Ricci L., Draghici E., Palagano E., Zonari E., Desantis G. (2021). Expanded circulating hematopoietic stem/progenitor cells as novel cell source for the treatment of TCIRG1 osteopetrosis. Haematologica.

[17] Chen J., Matatall K.A., Feng X., Hormaechea-Agulla D., Maharjan M., Young N., King K.Y. (2020). Dnmt3a-null hematopoietic stem and progenitor cells expand after busulfan treatment. Exp. Hematol..

[18] Peng M., Liao Q., Hu W., Tian G., Wang H., Zhang Y., Cheng R., Mamoulis N., Sun Y., Huang X. (2019). Pattern filtering attention for distant supervised relation extraction via online clustering. Web Information Systems Engineering–WISE 2019.

[19] Nakamura-Ishizu A. (2021). Thrombopoietin regulates mitochondria homeostasis for hematopoietic stem cell maintenance. Rinsho Ketsueki.

[20] Milsom M.D. (2019). Ex vivo expansion of functional hematopoietic stem cells, facilitating transplantation in the absence of conditioning. Hemasphere.

[21] Buza-Vidas N., Antonchuk J., Qian H., Månsson R., Luc S., Zandi S., Anderson K., Takaki S., Nygren J.M., Jensen C.T. (2006). Cytokines regulate postnatal hematopoietic stem cell expansion: Opposing roles of thrombopoietin and LNK. Genes Dev..

[22] Zhou Y., Zhu X., Dai Y., Xiong S., Wei C., Yu P., Tang Y., Wu L., Li J., Liu D. (2019). Chemical cocktail induces hematopoietic reprogramming and expands hematopoietic stem/progenitor cells. Adv. Sci..

[23] Ema H., Nakauchi H. (2000). Expansion of hematopoietic stem cells in the developing liver of a mouse embryo. Blood.

[24] Sauvageau G., Iscove N.N., Humphries R.K. (2004). In vitro and in vivo expansion of hematopoietic stem cells. Oncogene.

[25] Dahlberg A., Delaney C., Bernstein I.D. (2011). Ex vivo expansion of human hematopoietic stem and progenitor cells. Blood.

[26] Chatterjee C., Schertl P., Frommer M., Ludwig-Husemann A., Mohra A., Dilger N., Naolou T., Meermeyer S., Bergmann T.C., Alonso Calleja A. (2021). Rebuilding the hematopoietic stem cell niche: Recent developments and future prospects. Acta Biomater..

[27] Espinoza J.L., Kamio K., Lam V.Q., Takami A. (2021). The impact of NLRP3 activation on hematopoietic stem cell transplantation. Int. J. Mol. Sci..

[28] Ho Y.-H., Toro R.D., Rivera-Torres J., Rak J., Korn C., García-García A., Macías D., González-Gómez C., Monte A.D., Wittner M. (2019). Remodeling of bone marrow hematopoietic stem cell niches promotes myeloid cell expansion during premature or physiological aging. Cell Stem Cell.

[29] Dzierzak E., Bigas A. (2018). Blood development: Hematopoietic stem cell dependence and independence. Cell Stem Cell.

[30] Bhatia M., Bonnet D., Kapp U., Wang J.C., Murdoch B., Dick J.E. (1997). Quantitative analysis reveals expansion of human hematopoietic repopulating cells after short-term ex vivo culture. J. Exp. Med..

[31] Gao X., Zhang D., Xu C., Li H., Caron K.M., Frenette P.S. (2021). Nociceptive nerves regulate haematopoietic stem cell mobilization. Nature.

[32] Hétu-Arbour R., Tlili M., Bandeira Ferreira F.L., Abidin B.M., Kwarteng E.O., Heinonen K.M. (2021). Cell-intrinsic Wnt4 promotes hematopoietic stem and progenitor cell self-renewal. Stem Cells.

[33] Li J., Zhang L., Yin L., Ma N., Wang T., Wu Y., Wang M., Yang X., Xu H., Hao C. (2019). In vitro expansion of hematopoietic stem cells by inhibition of both GSK3 and p38 signaling. Stem Cells Dev.

[34] Ho Y.H., Méndez-Ferrer S. (2020). Microenvironmental contributions to hematopoietic stem cell aging. Haematologica.

[35] Yin X., Hu L., Zhang Y., Zhu C., Cheng H., Xie X., Shi M., Zhu P., Zhao X., Chen W. (2020). PDGFB-expressing mesenchymal stem cells improve human hematopoietic stem cell engraftment in immunodeficient mice. Bone Marrow Transplant..

[36] Abou-Ezzi G., Supakorndej T., Zhang J., Anthony B., Krambs J., Celik H., Karpova D., Craft C.S., Link D.C. (2019). TGF-β signaling plays an essential role in the lineage specification of mesenchymal stem/progenitor cells in fetal bone marrow. Stem Cell Reports.

[37] Araki D., Fu J.F., Huntsman H., Cordes S., Seifuddin F., Alvarado L.J., Cheruku P.S., Cash A., Traba J., Li Y. (2021). NOTCH-mediated ex vivo expansion of human hematopoietic stem and progenitor cells by culture under hypoxia. Stem Cell Reports.

[38] Sun Q., Fu Y., Zhu X., Tan W.S., Cai H. (2021). Continuous NF-κB pathway inhibition promotes expansion of human phenotypical hematopoietic stem/progenitor cells through metabolism regulation. Exp. Cell Res..

[39] Antonchuk J., Sauvageau G., Humphries R.K. (2002). HOXB4-induced expansion of adult hematopoietic stem cells ex vivo. Cell.

[40] Ohta H., Sekulovic S., Bakovic S., Eaves C.J., Pineault N., Gasparetto M., Smith C., Sauvageau G., Humphries R.K. (2007). Near-maximal expansions of hematopoietic stem cells in culture using NUP98-HOX fusions. Exp. Hematol..

[41] Chen Z., Guo Q., Song G., Hou Y. (2022). Molecular regulation of hematopoietic stem cell quiescence. Cell Mol. Life Sci..

[42] Ran Q., Guo C., Sun C., Liu Q., He H., Zhao W., Zhang J., Xiao Y. (2021). Loss of FGFR3 accelerates bone marrow suppression-induced hematopoietic stem and progenitor cell expansion by activating FGFR1-ELK1-cyclin D1 signaling. Transplant. Cell Ther..

[43] Fiegel H.C. (2006). Fetal and adult liver stem cells for liver regeneration and tissue engineering. J. Cell. Mol. Med..

[44] Bhardwaj R., Kumar L., Chhabra D., Mehra N.K., Sharma A., Mohanty S., Kochupillai V. (2021). In vitro expansion of fetal liver hematopoietic stem cells. Sci. Rep..

[45] Li H., Pei H., Xie X., Wang S., Jia Y., Zhang B., Fan Z., Liu Y., Bai Y., Han Y. (2019). Liver sinusoidal endothelial cells promote the expansion of human cord blood hematopoietic stem and progenitor cells. Int. J. Mol. Sci..

[46] Zhou K., Hu C., Zhou Z., Huang L., Liu W., Sun H. (2009). Fetal liver stromal cells promote hematopoietic cell expansion. Biochem. Biophys. Res. Commun..

[47] Becker-Herman S., Rozenberg M., Hillel-Karniel C., Gil-Yarom N., Kramer M.P., Barak A., Sever L., David K., Radomir L., Lewinsky H. (2021). CD74 is a regulator of hematopoietic stem cell maintenance. PLoS Biol..

[48] Chen Y., Fang S., Ding Q., Jiang R., He J., Wang Q., Jin Y., Huang X., Liu S., Capitano M.L. (2021). ADGRG1 enriches for functional human hematopoietic stem cells following ex vivo expansion-induced mitochondrial oxidative stress. J. Clin. Invest..

[49] Chute J.P., Muramoto G.G., Fung J., Oxford C. (2005). Soluble factors elaborated by human brain endothelial cells induce the concomitant expansion of purified human BM CD34+CD38- cells and SCID-repopulating cells. Blood.

[50] Zhang C.C., Lodish H.F. (2005). Murine hematopoietic stem cells change their surface phenotype during ex vivo expansion. Blood.

[51] Comazzetto S., Shen B., Morrison S.J. (2021). Niches that regulate stem cells and hematopoiesis in adult bone marrow. Dev. Cell.

[52] Méndez-Ferrer S., Michurina T.V., Ferraro F., Mazloom A.R., Macarthur B.D., Lira S.A., Scadden D.T., Ma’ayan A., Enikolopov G.N., Frenette P.S. (2010). Mesenchymal and haematopoietic stem cells form a unique bone marrow niche. Nature.

[53] Marx-Blümel L., Marx C., Sonnemann J., Weise F., Hampl J., Frey J., Rothenburger L., Cirri E., Rahnis N., Koch P. (2021). Molecular characterization of hematopoietic stem cells after in vitro amplification on biomimetic 3D PDMS cell culture scaffolds. Sci Rep..

[54] Bai T., Li J., Sinclair A., Imren S., Merriam F., Sun F., O’Kelly M.B., Nourigat C., Jain P., Delrow J.J. (2019). Expansion of primitive human hematopoietic stem cells by culture in a zwitterionic hydrogel. Nat. Med..

[55] Aerts-Kaya F. (2021). Strategies to protect hematopoietic stem cells from culture-induced stress conditions. Curr Stem Cell Res. Ther.

[56] Miller C.L., Eaves C.J. (1997). Expansion in vitro of adult murine hematopoietic stem cells with transplantable lympho-myeloid reconstituting ability. Proc. Natl. Acad. Sci. USA.

[57] Shao L., Elujoba-Bridenstine A., Zink K.E., Sanchez L.M., Cox B.J., Pollok K.E., Sinn A.L., Bailey B.J., Sims E.C., Cooper S.H. (2021). The neurotransmitter receptor Gabbr1 reg.gulates proliferation and function of hematopoietic stem and progenitor cells. Blood.

[58] Budgude P., Kale V., Vaidya A. (2020). Mesenchymal stromal cell-derived extracellular vesicles as cell-free biologics for the ex vivo expansion of hematopoietic stem cells. Cell Biol. Int..

[59] Islami M., Payandeh Z., Abdolahinia E.D., Saburi E., Soleimanifar F., Kehtari M., Mortazavi Y., Nadri S., Darvish M. (2019). Fucosylated umbilical cord blood hematopoietic stem cell expansion on selectin-coated scaffolds. J. Cell Physiol..

[60] Mesquitta W.-T., Wandsnider M., Kang H., Thomson J., Moskvin O., Suknuntha K., Slukvin I.I. (2019). UM171 expands distinct types of myeloid and NK progenitors from human pluripotent stem cells. Sci. Rep..

[61] Himburg H.A., Muramoto G.G., Daher P., Meadows S.K., Russell J.L., Doan P., Chi J.-T., Salter A.B., Lento W.E., Reya T. (2010). Pleiotrophin regulates the expansion and regeneration of hematopoietic stem cells. Nat. Med..

[62] Wen R., Dong C., Xu C., Zhao L., Yang Y., Zhang Z., Chen Y., Duan L., Chen H., Yang Z. (2020). UM171 promotes expansion of autologous peripheral blood hematopoietic stem cells from poorly mobilizing lymphoma patients. Int. Immunopharmacol..

[63] Zimran E., Papa L., Hoffman R. (2021). Ex vivo expansion of hematopoietic stem cells: Finally transitioning from the lab to the clinic. Blood Rev..

[64] Chagraoui J., Lehnertz B., Girard S., Spinella J.F., Fares I., Tomellini E., Mayotte N., Corneau S., MacRae T., Simon L. (2019). UM171 induces a homeostatic inflammatory-detoxification response supporting human HSC self-renewal. PLoS ONE.

[65] Park G.-B., Kim M.-J., Vasileva E.A., Mishchenko N.P., Fedoreyev S.A., Stonik V.A., Han J., Lee H.S., Kim D., Jeong J.-Y. (2019). Echinochrome a promotes ex vivo expansion of peripheral blood-derived CD34 + cells, potentially through downregulation of ROS production and activation of the src-lyn-p110δ pathway. Mar. Drugs.

[66] Wang N., Yin J., You N., Yang S., Guo D., Zhao Y., Ru Y., Liu X., Cheng H., Ren Q. (2021). TWIST1 preserves hematopoietic stem cell function via the CACNA1B/Ca2+/mitochondria axis. Blood.

[67] Bonora M., Kahsay A., Pinton P. (2021). Mitochondrial cal.lc.cium homeostasis in hematopoietic stem cell: Molecular regulation of quiescence, function, and differentiation. Int. Rev. Cell Mol. Biol..

[68] Uslu M., Albayrak E., Kocabaş F. (2020). Temporal modulation of calcium sensing in hematopoietic stem cells is crucial for proper stem cell expansion and engraftment. J. Cell Physiol..

[69] Chou S., Chu P., Hwang W., Lodish H. (2010). Expansion of human cord blood hematopoietic stem cells for transplantation. Cell Stem Cell.

[70] Chavez J.S., Rabe J.L., Loeffler D., Higa K.C., Hernandez G., Mills T.S., Ahmed N., Gessner R.L., Ke Z., Idler B.M. (2021). PU.1 enforces quiescence and limits hematopoietic stem cell expansion during inflammatory stress. J. Exp. Med..

[71] Luo Y., Li L., Zou P., Wang J., Shao L., Zhou D., Liu L. (2014). Rapamycin enhances long-term hematopoietic reconstitution of ex vivo expanded mouse hematopoietic stem cells by inhibiting senescence. Transplantation.

[72] Fujino T., Goyama S., Sugiura Y., Inoue D., Asada S., Yamasaki S., Matsumoto A., Yamaguchi K., Isobe Y., Tsuchiya A. (2021). Mutant ASXL1 induces age-related expansion of phenotypic hematopoietic stem cells through activation of Akt/mTOR pathway. Nat. Commun..

[73] Yu Z., Yang W., He X., Chen C., Li W., Zhao L., Liu L., Liu J., Xie L., Zhang Y. (2022). Endothelial cell-derived angiopoietin-like protein 2 supports hematopoietic stem cell activities in bone marrow niches. Blood.

[74] Zhang C.C., Kaba M., Ge G., Xie K., Tong W., Hug C., Lodish H.F. (2006). Angiopoietin-like proteins stimulate ex vivo expansion of hematopoietic stem cells. Nat. Med..

[75] He Q., Hong M., He J., Chen W., Zhao M., Zhao W. (2020). Isoform-specific involvement of Brpf1 in expansion of adult hematopoietic stem and progenitor cells. J. Mol. Cell Biol..

[76] Zhang C., Fondufe-Mittendorf Y.N., Wang C., Chen J., Cheng Q., Zhou D., Zheng Y., Geiger H., Liang Y. (2020). Latexin regulation by HMGB2 is required for hematopoietic stem cell maintenance. Haematologica.

[77] Hu Q., Zhang Y., Lou H., Ou Z., Liu J., Duan W., Wang H., Ge Y., Min J., Wang F. (2021). GPX4 and vitamin E cooperatively protect hematopoietic stem and progenitor cells from lipid peroxidation and ferroptosis. Cell Death Dis..

[78] Hou L., Voit R.A., Sankaran V.G., Springer T.A., Yuki K. (2020). CD11c regulates hematopoietic stem and progenitor cells under stress. Blood Adv..

[79] Adelman E.R., Huang H.T., Roisman A., Olsson A., Colaprico A., Qin T., Lindsley R.C., Bejar R., Salomonis N., Grimes H.L. (2019). Aging human hematopoietic stem cells manifest profound epigenetic reprogramming of enhancers that may predispose to leukemia. Cancer Discov.

[80] Sun X., Cao B., Naval-Sanchez M., Pham T., Sun Y.B.Y., Williams B., Heazlewood S.Y., Deshpande N., Li J., Kraus F. (2021). Nicotinamide riboside attenuates age-associated metabolic and functional changes in hematopoietic stem cells. Nat Commun..

[81] Pereira C.F., Chang B., Qiu J., Niu X., Papatsenko D., Hendry C.E., Clark N.R., Nomura-Kitabayashi A., Kovacic J.C., Ma’ayan A. (2013). Induction of a hemogenic program in mouse fibroblasts. Cell Stem Cell.

[82] Sugimura R., Jha D.K., Han A., Soria-Valles C., da Rocha E.L., Lu Y.F., Goettel J.A., Serrao E., Rowe R.G., Malleshaiah M. (2017). Haematopoietic stem and progenitor cells from human pluripotent stem cells. Nature.

[83] Lee S.-J., Kim J.-H., Kang K.-W., Park Y., Kim B.-S. (2020). Supporting data on enhanced reprogramming of human CD34+ hematopoietic stem cells to induced pluripotent stem cells using human placenta-derived cell conditioned medium. Data Brief..

[84] Kunisaki Y., Bruns I., Scheiermann C., Ahmed J., Pinho S., Zhang D., Mizoguchi T., Wei Q., Lucas D., Ito K. (2013). Arteriolar niches maintain haematopoietic stem cell quiescence. Nature.

[85] Sandler V.M., Lis R., Liu Y., Kedem A., James D., Elemento O., Butler J.M., Scandura J.M., Rafii S. (2014). Reprogramming human endothelial cells to haematopoietic cells requires vascular induction. Nature.

[86] Riddell J., Gazit R., Garrison B.S., Guo G., Saadatpour A., Mandal P.K., Ebina W., Volchkov P., Yuan G.C., Orkin S.H. (2014). Reprogramming committed murine blood cells to induced hematopoietic stem cells with defined factors. Cell.

